# Factors influencing adherence to tuberculosis treatment in Asmara, Eritrea: a qualitative study

**DOI:** 10.1186/s41043-017-0132-y

**Published:** 2018-01-05

**Authors:** Frezghi Hidray Gebreweld, Meron Mehari Kifle, Fitusm Eyob Gebremicheal, Leban Lebahati Simel, Meron Mebrahtu Gezae, Shewit Sibhatu Ghebreyesus, Yordanos Tesfamariam Mengsteab, Nebiat Ghirmay Wahd

**Affiliations:** 1 Ministry of Health, Asmara, Eritrea; 2 Department of Epidemiology and Biostatistics, School of Public Health, Asmara College of Health Sciences, Asmara, Eritrea

**Keywords:** Tuberculosis, DOTS, Barriers to treatment adherence, Asmara, Eritrea

## Abstract

**Background:**

Non-adherence to tuberculosis (TB) treatment is an important barrier for TB prevention and control. Poor adherence may result in prolonged disease infectiousness, drug resistance, relapse and death. The aim of this study was to assess factors influencing adherence to tuberculosis treatment in selected health facilities in Asmara, Eritrea.

**Methods:**

A qualitative study which included in-depth interviews with 12 TB patients, three focus group discussions in selected health facilities in which one group comprised eight patients and key informant interviews with three health workers. Data analysis was done by translating and transcribing the verbatim of the interviews and focus group discussions. Transcribed data was then analysed using thematic framework procedure.

**Results:**

This study found that patients lacked knowledge about the cause, transmission and duration of treatment of TB. The most common reason mentioned for discontinuing treatment was the patient “felt cured”. Almost half of the respondents did not know the standard treatment duration and the consequences they face if they halt treatment. Patients reported losing their job when their diagnosis was known, were too ill to continue working or unable to find daily work due to time-consuming treatment arrangements. With few exceptions, the majority of patients reported that the short distance to the clinic encouraged them to attend regular treatment follow-up. Most of the respondents were unable to get enough food, leading to stress and feelings of hopelessness. Lack of social support for most of the patients was a critical factor for adherence as were stigma, medication side effects and long treatment duration. Recognized as an enabler to treatment adherence, health workers had good communication and positive attitude towards their patients.

**Conclusion:**

Lack of knowledge, loss of income, stigma and lack of social support, drug side effects and long treatment duration emerged as important barriers for treatment adherence. Short distances to health facilities, good communication and accepting attitude of health care providers emerged as enablers for treatment adherence. For better treatment adherence, comprehensive health education at treatment sites, patient’s family members and the community at large and strengthening of social support structures need to be addressed.

## Background

Despite being a curable disease, tuberculosis (TB) continues to remain a major global health problem [1]. TB is the second leading cause of death from infectious diseases worldwide and the leading preventable cause of death among people living with HIV [2]. In 2015, there were an estimated 10.4 million new TB cases and 1.4 million TB deaths, with an additional 0.4 million deaths resulting from TB among HIV-positive people. In the same year, the TB case fatality rate varied widely—from under 5% in some countries to more than 20% in most countries in the WHO African Region. This highlights the persistence of large inequities in access to high-quality diagnostic and treatment services, widespread poverty, poor treatment adherence and lowered immunity in these countries [3, 4]. This is particularly significant for the developing world because low- and middle-income countries share the largest burden of TB, worsened by the fact that about 75% of TB patients are in the youngest and economically most productive age groups (15–54 years) [3].

Adherence to TB treatment is crucial to avert disease infectiousness, achieve cure and avoid emergence of drug resistance, relapse and death. Non-adherence to TB treatment is an important barrier and is one of the most significant obstacles to TB control globally and it has become a major contributing factor for treatment failure [5]. In sub-Saharan Africa, there is high rate of losses to follow-up of TB patients that ranges from 11.3 to 29.6% [6].

Research shows that there are many interplaying factors influencing treatment adherence of TB patients. Lack of knowledge about TB in general and treatment regimen and length in particular, loss of employment or the opportunity to work and subsequent financial difficulties, transport problems and lack of access to health services, social stigma and discrimination, medication side effects, long treatment period, inadequate food, poor communication with service providers are some of the factors empirically shown to affect drug adherence [7–9]. Knowledge of these factors helps to orient future actions taken within the framework of the National TB Control Program with the purpose of containing the heightened rate of therapeutic failure, the increase of the circulation of drug-resistant strains, and therefore improving the overall control of the disease.

In Eritrea, the current health facility-based directly observed treatment schedule (DOTS) coverage is almost universal. Despite this extensive service coverage, however, the program performance indicators still remain unsatisfactory [10, 11]. The morbidity rate of TB increased from 97/100,000 in 2011 to 123/100,000 in 2014, while the mortality rate increased from 12/100,000 in 1990 to 20/100,000 in 2014. Moreover, there were 39 treatment failure cases in 2014 and 44 cases in 2016. The WHO Global TB Report also stated that the multi-drug-resistant (MDR) TB rate was estimated to be 1.8% among new cases and 19% among previously treated cases in Eritrea [12]. This indicates that the TB control program cannot be successful with extensive service availability alone. Thus, successful treatment outcomes primarily require a better and deeper understanding of the barriers and enablers the patients experience during the whole treatment period. However, relatively few qualitative studies have tried to assess the factors acting as barriers and enablers of TB treatment adherence and this has yet to be studied in Eritrea. In this context, this study was carried out as an initial attempt to assess the factors influencing adherence to TB treatment in three health facilities of Asmara, Eritrea. The findings may serve as baseline data for further research and inform policy and strategic implementation for effective treatment of TB in Eritrea.

## Methods

### Study design

This study used a qualitative study design to identify factors influencing adherence to treatment among TB patients in Asmara, Eritrea.

### Study sites

Six health facilities were designated as DOTS giving sites in Asmara by the Ministry of Health. Three health facilities were selected using a simple random sampling technique. The selected health facilities were Villajo Community Hospital, Akria Health Centre and Godaif Community Hospital.

### Sampling method

A purposive sampling technique was used to select all study participants. Twelve patients (seven females and five males) participated in the in-depth interviews (i.e. four respondents from each health facility). Focus group discussions were arranged in each study site with eight TB patients in each group. Three health personnel (one from each study site) assigned to the TB outpatient department were also interviewed as key informants.

### Data collection instruments and techniques

We developed a questionnaire, subsequently translated to the local language (Tigrinya), from an extensive review of the literature. Open-ended questions were used to identify the potential factors that influence adherence to TB treatment.

For better comprehension of the factors affecting treatment adherence, this study used triangulation by conducting in-depth interviews, focus group discussions and key informants. The research team conducted face to face tape-recorded, in-depth interviews which took 30–45 min on average, focus group discussions for about 90 min each and key informant interviews. Each FGD session was accompanied by one moderator, one note taker and a recorder. All interviews took place at locations chosen by the respondents. Notes were also taken at the time of interview to aid researchers in memorizing each of the respondents’ interview.

### Data analysis procedure

Interviewer-related bias was addressed by continuously discussing and negotiating the content of key words, broader concepts and units of meanings. Then, the researchers discussed and clarified the content of each tape-recorded interview. The notes enabled the researchers to read about each of the participants that were interviewed and quoted in the document. Next, the verbatim of each interview and discussions in the focus group discussions were translated and transcribed. A list of all themes was prepared and written next to appropriate segments of the transcripts. Transcribed data was manually analysed by using thematic framework procedures which involved four main stages: familiarization, identifying thematic framework, coding and interpretation.

The data was summarized in the analysis document that all of the research team commented. The results were presented to the research team to ensure that the experiences of the study participants were accurately captured and reflected.

### Ethical considerations

The ethical clearance for conducting the study was granted by Asmara College of Health Sciences, School of Public Health Ethical clearance committee. After brief explanation of the purpose of the study, written consent was obtained from the study participants. Confidentiality was assured by excluding personal identifiers. Participants had the right to withdraw from the study at any time.

## Results

A total of 39 participants were successfully interviewed, of which 20 were females and 19 males. The mean age of the participants was 37 years (range 18 to 61 years) (Table 1).

**Table 1:** Demographic characteristics of the respondents

	Number
Age	
20–30	14
30–40	11
40–50	7
50+	7
Educational level	
No education	9
Elementary school	8
Junior	6
High school	6
College level	10
Participant category	
In-depth interview (IDI)	12
Focus group discussion (FGD)	24
Key informant (KI)	3

From the content analysis, six sub-themes were developed. These were comprehensive knowledge about TB, transportation, loss of source of income (loss of employment), social factors, drug side effects and long period of treatment and communication with health care provider.

### Comprehensive knowledge about tuberculosis

In this study, the respondents lacked general knowledge regarding TB. Most of the patients had no or an ambiguous idea as to what caused the disease, the mode of transmission or length of treatment duration.

I always wake up at five in the morning for work in the garden when it’s so cold, which caused the disease. (47-year-old male farmer, FGD)

When respondents were asked how they felt once they first knew about their illness, some of them confused TB with AIDS at the time of diagnosis, considered TB as a hereditary disease while some respondents did not consider TB to be a contagious disease or did not know its mode of transmission.

When I was told by the doctor that I had TB, I was totally shocked. I’ve heard that a long stay with TB will eventually lead to AIDS. I was very frustrated because as a house wife, I have never gotten any injury with sharps to explain in case of AIDS. (43-year-old female, in-depth interview)

Well........ I don’t really know how I got this disease, no one had gotten this disease in our family before and we don’t have its trait. (53-year-old female, in-depth interview)

During the interviews, participants mentioned that they were not provided with regular health education at the health facilities. On focus group discussion, one respondent explained:

We are not provided with regular health education regarding tuberculosis. The nurse just advices us to attend daily and avoid being late. If you are lucky you get information when you go to Godaif for follow up in two months. (41-year-old female, FGD)

A 25-year-old key informant who worked 5 years as a TB focal person shared her experience about the knowledge of patients regarding TB and its treatment at the time of diagnosis.

I have worked in the TB clinic as a focal person for 5 years. Most of the patients had little knowledge about the disease and they easily get scared once they know about the disease. Based on my experience, I feel that the community also has very little knowledge. (25-year-old female, KI)

The most common reason mentioned for discontinuing treatment by both respondents and key informants was that the patient “felt cured”. Almost half of the respondents did not know the standard treatment duration was 6 months and what the consequences would be if they discontinue the treatment.

A female participant who had defaulted in the fifth month and was on retreatment commented on why she did not complete the treatment regimen.

I did not know about the risks of stopping the treatment. I took the medication for five months; my weight became normal and I felt healthy. That’s all I wanted. So I simply stopped going to the clinic or taking the medication. (22-year-old female, FGD)

### Transportation

Majority of the participants reported that distance to the clinic acted as enabler to follow their treatment regimen. At the time of diagnosis, patients were allowed to choose a clinic to take their medications which is the closest to them.

I walk only 300 meters to get to the clinic; this distance helped me to follow my treatment. If my house was in Mai-Anbesa (900 metres away), no doubt I would have stopped the treatment. (24-year-old male, in-depth interview)

However, few patients from villages surrounding Asmara claimed transportation as a barrier. They reported that there were no DOTS giving clinics or no means transportation in their villages and had to go to the next nearest village. Few patients stated that even with the availability of transport services, its daily expenses for 6 months is challenging.

A 51-year-old female who lives in Embeyto (a village 20 km from Asmara), during FGD, commented:

I am from Embeyto. When I first knew about my disease, I decided to take the daily medication from Adi Hawesha but it was mountainous and I decided to change to AdiHakefa. For three weeks, I walked two hours daily and used to rest many times. Because I was completely exhausted, I had to search for a house in Asmara. Luckily, my niece gave me a small room in Godaif from where I still continue to follow my treatment. (51-year-old female, FGD)

Another participant from an in-depth interview explained:

I am from Adi Guaedad, I was informed to take medication from Merhano. This means I had to do a one hour walk daily. I was reluctant because it was difficult and I opted to go to Godaif. It is still a long distance and, there is lack of transportation. Sometimes I use taxi but it is so expensive. I can’t afford it. (37-year-old female, in-depth interview)

### Loss of source of income (loss of employment)

The majority of the patients experienced loss of employment or the opportunity to work as one of their main problems during treatment. Patients reported losing their job when their diagnosis was known, were too ill to continue working, or were unable to find daily work because of the time consuming treatment arrangements. One respondent who claimed to have lost her job due to TB explained her condition during focus group discussion as follows:

I worked in Merhano’s church as gate keeper. I lost my job because of the disease as I was too weak to work. The doctor told me that the treatment lasts for six months and I had no one to help me as my condition got worse. After a long struggle, I found my grandson who lives in Asmara. Even though he is economically unstable, I decided to stay with him until completion of the treatment. (62-year-old female, FGD)

A 43-year-old male participant also remarked:

I work as a daily labourer and I lost my job because I was feeling weak and had severe joint pains. I got admitted in the hospital for one month before starting medication. At that time, I spent all my finances. I now depend on families and friends for support, which makes it so difficult for me to focus and continue on my treatment. (43-year-old male respondent, in-depth interview)

Impoverishment due to loss of income caused unpredictability and emotional stress in relation to daily access to food. Most patients stated that they suffered a lot from shortage of food. A female respondent from the in-depth interview, who lives alone and was on the fifth month of her treatment described:

In the first three months, I used to eat eggs, milk and meat because I could afford them. I stopped later on because of the increasingly high expenses. My health was then immediately deteriorated. At times, I experienced shivering and staggering. (51-year-old female, in-depth interview)

A 24-year-old male respondent during FGD explained the importance of food as follows:

Food is very important. If the clinic could provide us with food aid, it would be supportive. Medication without food is nothing. I would rather get food without medication. (24-year-old male, FGD)

During the interviews, key informants mentioned that shortage of food as key factor for treatment non-compliance.

One of the main problems TB patients encounter is lack of food, we advise them to eat protein rich food if they can afford it but the reality is they can’t. I remember in previous years governmental organizations like National Union of Eritrean Youth and Students (NUEYS) provided milk patients, but now this is no longer the case. If these initiatives resume and other NGO’s start to provide support, the problem could be reduced. (46-year-old female, KI)

### Social factors

In this study, the social context which includes family, community and household support, including stigma, emerged as important factors influencing adherence to treatment.

### Social isolation (stigma)

Many patients believed that they were predisposed to stigma because of the disease. Some shared their experiences, such as being pointed at in their neighbourhoods, neighbours gossiping about their illness and exclusion from social events. These actual experiences and perceived stigma resulted in many patients hiding their diagnosis or only disclosing it to selected people, mostly to close friends and families. Some patients were reluctant to go to clinic because they were too afraid of being recognized by neighbours. This concern was also mentioned by health workers. During in-depth interviews the following comments were given:

I feel uneasy while going to the clinic. In case someone sees me, I wait until everybody has taken their medication and went. (26-year-old female, in-depth interview)

Even if people are doing their normal activities I feel that they are looking at me. When I come every day carrying the water bottle I feel ashamed so I hide it in my bag. (48-year-old female, in-depth interview)

A 26-year-old health worker who worked as a TB focal person for 3 years shares her experience on this:

Three months ago, I had a TB patient who said that the community fears TB more than AIDS. She told me that if the neighbours happen to know that she had TB, the house owner would force her to leave the house. (26-year-old female, KI)

Some of the patients commented that stigma starts from the family. According to them, the fact that the disease is contagious forces patients to have separate utensils and a separate bed room. The family members also try to restrain their contact due to the fear of acquiring the disease. The respondents claim that these seemingly minor actions were serious enough to cause them frustration and additional stress.

One participant from the FGD remarked:

In the first three months, I lived in an isolated room. I had poor contact with my wife and children. I stayed on my own day and night and it was very stressful. Sometimes I wondered about the meaning of life itself. (50-year-old male, FGD)

### Social support

This study revealed that family and community support are extremely important factors during treatment, because, to a large extent, they compensate for loss of income. Many of the patients received physical support in walking to the clinic and some, money for transportation. Respondents in the FGD described:

I have no strength to carry water and perform other physical activities. My neighbours help me. My former employer has also helped me this far. (35-year-old female, FGD)

One respondent in the in-depth interview described:

I don’t have any job. I get financial support from my brothers who live abroad, may God help them. (26-year-old female, in-depth interview)

Lack of social support was found to be one of the main barriers to TB treatment adherence. Around half of the patients in this study did not receive any type of social or financial support from their family or community. Many patients experienced changes in the level of support as the treatment program progressed.

Respondents from in-depth interview explained:

People are sympathetic ......... act sympathetic, but just in words. No one came forward to help me financially. (29-year-old female participant)

One respondent from in-depth interview explained:

I live with my mother who is physically disabled. I am the sole bread winner and when I got sick I stopped working. After taking medication for three weeks only, I was already in a financial crisis. I wonder how I will cope till the end. (22-year-old male, in-depth interview)

### Drug side effects and long period of treatment

Most respondents mentioned that they experienced side effects from the treatment, manifested as nausea, vomiting, joint pain, and felt sleepy or too weak to walk. Some of the patients even believe that the treatment worsened their condition. Participants from in-depth interview said:

Oh! The medication made me very sick! I couldn’t control my balance and I got very tired to go to the clinic. Sometimes I thought of stopping the treatment. (37 years old female, in-depth interview)

At first I heard that TB medication causes gastritis. Currently I am on HIV medication and I can’t tolerate all the pills. I thought of stopping the medicine but my husband encouraged me to continue. (33-year-old female, in-depth interview)

Other participants from the FGD said:

Whenever I take the pills I feel joint pain, gastritis and feel sleepy. There are days when I slept until midday and missed the clinic appointment. (24-year-old male, FGD).

When a key informant was asked about medication side effects, she replied:

Vitamin B-complex prevents anorexia and protein helps to those who are underweight patients. But we can not provide these supplements for all of them. (26-year-old female)

Some participants complain that the treatment period is too long and leads them to different problems. A 38 years old male from the in-depth interview commented:

If possible the length of treatment has to be reduced. I heard it was eight months and now they changed it to six months. It’s good to reduce it further. (38-year-old male, in-depth interview)

### Communication with health personnel

The hospitability received by health care providers, including whether effective communication takes place, appeared to have a major impact on the patient’s adherence. The majority of patients in the study were happy on the way health professionals received and treated them at the health centres. Participants from the FGD commented the following:

I can not explain the nurse with words, she is like an angel. She knows our feelings without saying a word. Our relation is so close and intimate. She really encourages and inspires us to complete the treatment. (26-year-old male, FGD)

An 18-year-old student who dropped out from school due to TB commented during FGD:

In the first two weeks of the treatment, I was unable to withstand the drug side effects. I wanted to stop the medication and continue my education. But the health personnel counselled me and helped me change my mind. I am now convinced that my health is more important than anything else, and the drug side effects are temporary. I have no words to thank her and I am deeply indebted to her. (18-year-old female, FGD)

A key informant commented on the way she treats her patients:

It is my duty to take care of my patients and I try to help them as much as I can. For example, if they are disabled or in any way can not come to the clinic I allow them take the medication from health promoter. I do not want my patients to miss even a single day. In some cases when they are late or absent, I take the medication to their homes.

## Discussion

This study provided baseline information on factors that influence TB treatment adherence and found that several factors play as barriers to adherence. These were lack of general knowledge regarding TB, loss of income, stigma and lack of social support, drug side effects and long treatment duration. Factors such as short distances to health facilities and good communication and accepting attitude of health care providers were found to be enablers for treatment adherence.

We found that the majority of the respondents lacked adequate knowledge about TB. Most of the respondents did not know the actual cause, mode of transmission and treatment length. Some patients believed TB as a hereditary and deadly disease which has no cure. A number of respondents did not know the conventional treatment period is 6 months and the risk they face if they stop the medication. This was similar to study findings from Pakistan [13], Uganda [14], Nepal [15] and Papua New Guinea [16]. This shows that patients were not provided with enough health education while taking their medication. Studies have found that educating a TB patient significantly reduces the risk of treatment non-adherence [5, 6]. Hence, health care providers should be trained and encouraged to provide a more personalized health education within the context of the patient’s background and local customs.

Most of the respondents were encouraged to take their treatment properly because distance to health facility was relatively manageable. The Ministry of Health addresses distance barriers by training TB promoters from the community to provide medication to those who are unable to reach the clinic. Although distance was a minor concern, some patients around Asmara still faced a problem. Some participants had to walk for about 2 h a day to reach the clinic. Similar studies report that distances from patients’ homes to the health facilities and financial burdens contribute to diagnostic delay and treatment non-adherence [17], which discourage patients for treatment initiation and compliance [18].

This study revealed that few patients incurred high costs for transport to reach the clinic. Participants indicated that these problems make it difficult to take TB treatment readily because they are unemployed and their family or relatives cannot always provide them with financial support. A similar study found that TB patients experience problems concerning shortage of funds for transport because they are too weak to reach the facility on foot [19, 20]. Therefore, further decentralization of clinics which provide DOTS is necessary. This study also showed that illness-induced poverty over time threatens the sustainability of personal finances. Most of the respondents lost their source of income, which made them financially dependent. A similar study from China showed that economic burden might act as a barrier and indicated that loss of employment together with extra food demand and transport expenses over time cause a serious financial problem to patients and their immediate family [21]. The finding of this study points that even though free TB treatment is available, financial problems can still affect treatment adherence (e.g. costs of transportation). The WHO-TB treatment guideline recommends that patients may receive incentives in order to encourage them to be compliant with treatment [22]. However, the respondents reported that they do not receive any incentives and suggest it would be supportive if they were provided with some form of assistance.

The TB medication demands patients to consume extra food especially protein-rich foods to restore their health, which often goes beyond their financial ability [23]. From this study, it is clear that lack of food is an important barrier to treatment adherence. In a similar study, Mabunda and Bradley state that lack of food was reported to be the main barrier for treatment adherence because “one cannot take treatment on an empty stomach” [24]. To address this issue, collaboration with different sectors would be helpful, such as liaising with governmental and non-governmental organizations for providing food aid to patients.

Previous studies have shown that family and community support act as a main enabler for treatment adherence [25–27] and lack of access to such support systems as one of the major causes of non-adherence among TB patients [28]. In this study, the majority of the patients did not receive social or financial support from their family or their community. Many patients reported that they were forced to seek support due to loss of income and physical exhaustion. The respondents underscored that any sort of support, especially from family members, was a crucial factor to continuing their treatment. This suggests that patients, who have fewer human or material resources available, such as the very poor, single mothers or the elderly, do not benefit from such protective factors.

Stigma related to TB was an evident factor stated by many participants of this study as a main obstacle while taking medication. Patients were reluctant to take medication in the health facility because they feared being recognized by their neighbours. As indicated in the results, patients do not disclose their disease to anyone for practical reasons. For instance, some patients posited that if their house owner came to know about their sickness, the patient would be expelled from the house.

From the patients’ responses, experiences of drug side effects made some of them believe that the treatment was worsening their condition. This could mean that TB patients should be given medication to curb the side effects besides their TB treatment or at least be informed of the commonly anticipated side effects and the things to do when they occur.

Prolonged duration of TB treatment affects patients’ adherence as it affects their daily routine activities. This causes challenges for the patients and their families because it exhausts them financially, physically and psychologically. Similar studies have found that the lengthy course of TB challenges both patients and their families financially and emotionally, resulting in non-adherence to treatment [29, 30]. This might indicate that the TB treatment protocol needs to be more flexible, so that the treatment schedule is more convenient to patients, allowing them to schedule clinic visits at convenient time. It is also important to include family members and relatives in the management plan of the patient so that they understand and can know what to expect during the whole treatment period.

This study found that health professional’s interaction and their positive attitudes towards the patients were optimal. Patients reported that health workers’ communication and positive attitudes were a source of motivation to patients to stick with the treatment. Studies have noted that social events and obligations like illness of relatives and attendance at funerals or marriages were one of the most frequently cited reasons for treatment interruption [31]. In such instances, health care providers supply the medicine to their patients for up to 2 to 3 days with full information. This reduces the number of patients who interrupt treatment to attend social events.

This study has assessed factors influencing adherence to tuberculosis treatment in selected health facilities in Asmara, Eritrea. However, the results of this study should be interpreted with caution. Although the findings of the study may shed light to the current situation, they may not be generalized to the whole TB patient population in Eritrea. As the study was restricted to one geographical area and the qualitative nature of the study, the findings may not be transferable to other contexts or studies that utilize quantitative study design. Yet, the findings enable researchers to understand the broad contextual frame of potential similarities in similar contexts, in particular how certain identifiable patterns may act as enabling factors or create barriers to TB treatment adherence. Further, nationwide, preferably quantitative, studies should be conducted to broaden the current understanding of the factors associated with TB treatment adherence in Eritrea.

## Conclusion

This study showed that inadequate general knowledge on TB, loss of employment, stigma and lack of social support, medication side effects and long treatment period posed as barriers to treatment adherence. The short distance to reach health facilities and good communication and positive attitude of health care providers towards their patients were found to be enablers to treatment adherence. For better treatment adherence, comprehensive health education at treatment sites, patient’s family members and the community at large and strengthening of social support structures need to be addressed.
